# Corrugated Sheeting as a Member of a Shear Panel Under Repeated Load—Experimental Test

**DOI:** 10.3390/ma13184032

**Published:** 2020-09-11

**Authors:** Natalia Korcz-Konkol, Piotr Iwicki

**Affiliations:** Department of Metal Structures, Faculty of Civil and Environmental Engineering, Gdańsk University of Technology, ul. G. Narutowicza 11/12, 80-233 Gdańsk, Poland; piotr.iwicki@pg.edu.pl

**Keywords:** steel structures, stressed-skin effect, diaphragm design, trapezoidal sheeting, condition assessment of steel structure, structural health monitoring

## Abstract

In stressed-skin design, the cladding stiffening effect on structures is taken into account. However, the “traditional” design is more usual, wherein this effect is neglected. Even if the diaphragm actions are not regarded, in particular cases such as big sheds (and others), the parasitic (unwanted) stressed-skin action may occur with the result of leakage or even failure. The structures of this kind have already been built. Thus, an important question arises: How can one assess them if there is a need to correct or redesign them? What kind of non-destructive approach can be used to achieve that? Experimental tests of small-scale shear panels made of trapezoidal sheeting were designed in order to observe the behaviour of the diaphragm under increasing and repeated load. The tests were oriented toward force–displacement relations and strains in selected areas of the sheeting. The results revealed nonlinear, hysteretic force–displacement behaviour of the panel and the occurrence of the persistent deflections and stresses which remain even after the unloading. The relation among the stresses, force–displacement paths and modes of failure can be potentially used in monitoring systems of existing buildings in terms of parasitic stressed-skin action.

## 1. Introduction

Cladding of steel structures affects the stiffness and spatial character of structural performance, and consequently, deflections and forces in particular structural members. This action is called stressed-skin or diaphragm action. The idea of stressed-skin design was born in the 1960s, and came to be widely known throughout the decades. The intention is to take advantage of the interaction between structural elements and panels (roofing, wall cladding or even flooring). According to [1], roof and floor panels may be treated as the web and edge members (elements along the structure, e.g., purlins) as the flanges of the deep plate girder. Similarly, wall diaphragms may be treated as bracing. That is typical response of the stressed-skin structure on the horizontal forces (such as wind loads); however, in the case of a pitched-roof, vertical forces (such as snow loads) may be resisted in a similar way as well.

Recent European Recommendations for stressed-skin design [2] were formulated in 1995. Since then, significant changes were noted in steel construction: nowadays the structures are bigger, taller, made of more slender members (e.g., cold-formed profiles), have new types of cladding and fasteners, etc. What is more, the development of hardware and numerical software dedicated to structures has brought in more and more accessible tools to consider more complex cases. These are probably some of the reasons for the increasing interest of the researchers in stressed-skin actions. The improvements to the analytical procedures are investigated, e.g., new factors affecting stiffness of the diaphragms but neglected in the procedures and analyses of schemes of the fasteners, e.g., the lack of seam fasteners [3,4,5,6], and schemes known from practice but not included in recommendations [7,8]. Some of the necessary improvements of design codes have been proposed earlier [9]. Other studies focus on numerical aspects of taking into account the diaphragm actions, such as [8,10,11,12].

Simultaneously, in “traditional design” stabilisation of particular elements, e.g., purlins, by sheeting is much more eagerly taken into account (this problem was analysed, e.g., in [13]), while the diaphragm effect is neglected; thus, extra global stiffening of the structure by sheeting is considered beneficial. However, it has to be remembered that regardless of possible considering the stressed-skin actions of the cladding, it brings extra structural stiffness to a certain extent. The significance of this aspect is controlled by many parameters. In [14,15] the attention was drawn to selected roof failures attributed to the parasitic (undesired) stressed-skin action. Further discussion and explanations are presented also in [16]. The unwanted diaphragm actions may trigger significant force increases in the edge members (e.g., purlins), purlin to cleat connections, purlin to cladding connections and cladding itself. These situations arise in big sheds, but can be also important in small buildings, e.g., with cold-formed structure of the frames. The problem is even more complex because of the fact that buildings may be susceptible to fatigue accumulation deficits, reaching failure conditions many years after their construction, possibly causing leakage of the cladding, and even fatigue failure. These alarming reports should lead to much more cautious design of steel structures in the traditional approach.

The problem of the underestimation of the importance of the diaphragms is known and actual not only in steel structures. Another example may be the infilled reinforced concrete (RC) structures when subjected to earthquakes. According to [17], the infill masonry walls have a significant contribution to the global seismic response of RC structure. Results of the blind test prediction carried out in a scaled RC structure confirmed that the strength and stiffness of the infill (diaphragm) should definitely be taken into account in design processes as a very important factor.

A list of problems to solve thusly arose. How can one evaluate the condition of the structure in the existing buildings designed without the consideration of stressed-skin effect, currently considered as being in the group of risk of parasitic stressed-skin action? How can one assess them if there is a need to correct or redesign the structure before the leakage or failure occurred? What kind of non-destructive approach can be used to achieve this?

This paper attempts to address the issues above. Experimental tests of small-scale shear panels made of trapezoidal sheeting were designed in order to observe the behaviour of diaphragms under increasing and repeated load. The measurement results were the force–displacement relation (in a testing machine) and strains in selected areas of the sheeting using strain gauges (SG). The results revealed the hysteresical character of panel work and persistent displacements and strains which remain even after unloading the structure, which can be the first step to leakage, even failure. The outcomes showed the relation between the stresses, force–displacement paths and the mechanism of failure, to be potentially applied in the system of monitoring existing buildings in terms of parasitic stressed-skin action.

## 2. Materials and Methods

### 2.1. Experimental Model—General Description

**Figure 1:**
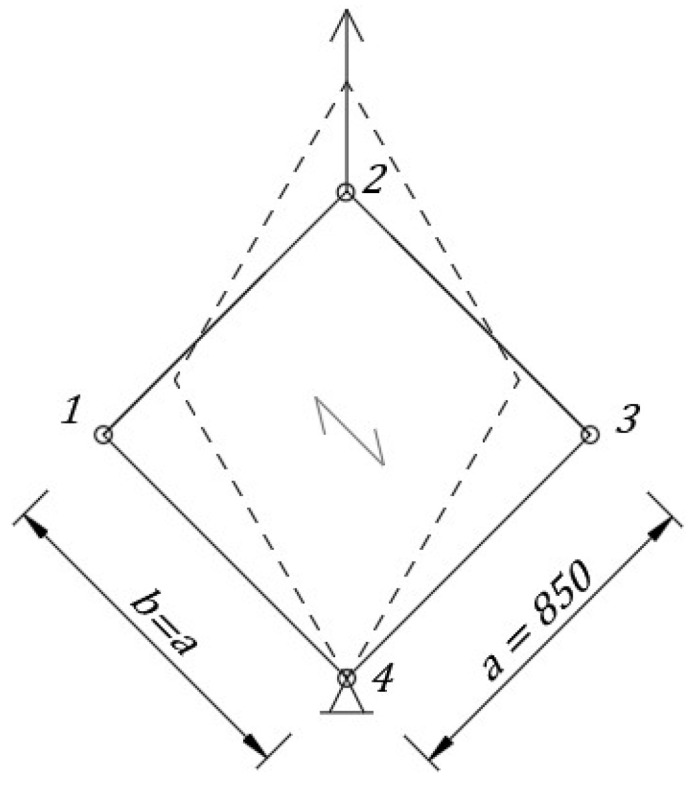
Static scheme of the experimental set-up [mm].

**Figure 2:**
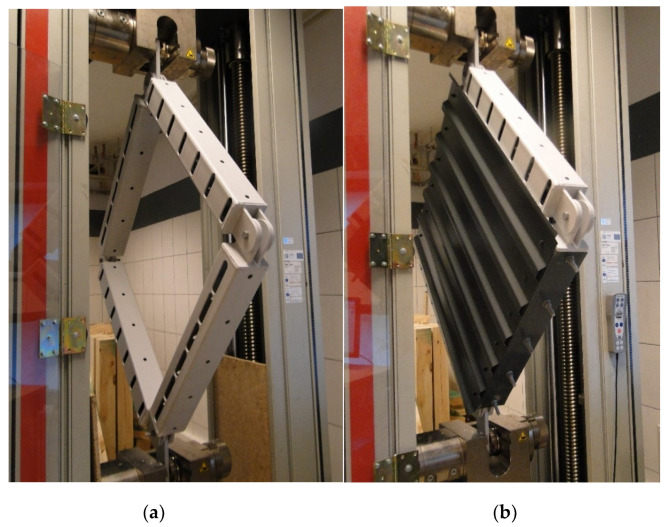
Experimental set-up: (**a**) permanent elements, (**b**) set-up with the analysed sheeting.

Experimental research was intended to investigate a wide range of panel behaviour at shear. The experimental set-up was designed for the corrugated sheet investigation; however, it can also be used for other types of panels (e.g., sandwich, wood or textile panels or parts of roofs consisting of purlins and trapezoidal sheeting). A static model of the permanent set-up is presented in Figure 1, the main elements are shown in Figure 2. Four pin-ended rectangular hollow section members form a square frame with four hinged nodes in the frame plane. The axial dimension of the frame was 850 mm (width and length: *a* and *b* in Figure 1). The dimensions of the set-up were assessed according to the technological condition that the largest dimension in the direction of square frame diagonal must be less than 1350 mm, i.e., the maximum allowable spacing between the fastening elements of the testing machine.

**Figure 3:**
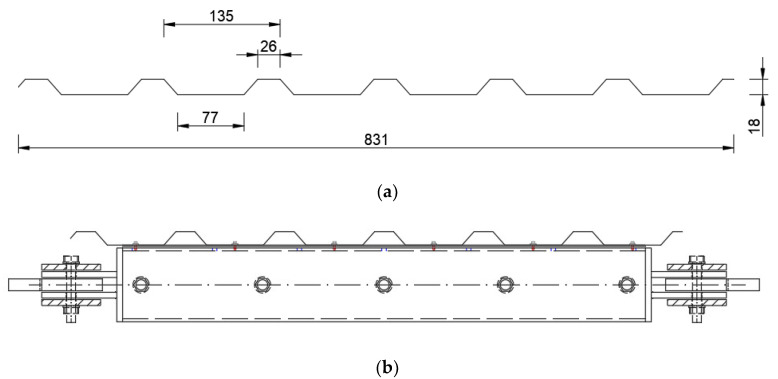
Specimens’ details: (**a**) trapezoidal sheeting geometry [mm]; (**b**) trapezoidal sheeting fastened to the permanent frame (scheme of the fasteners).

**Figure 4:**
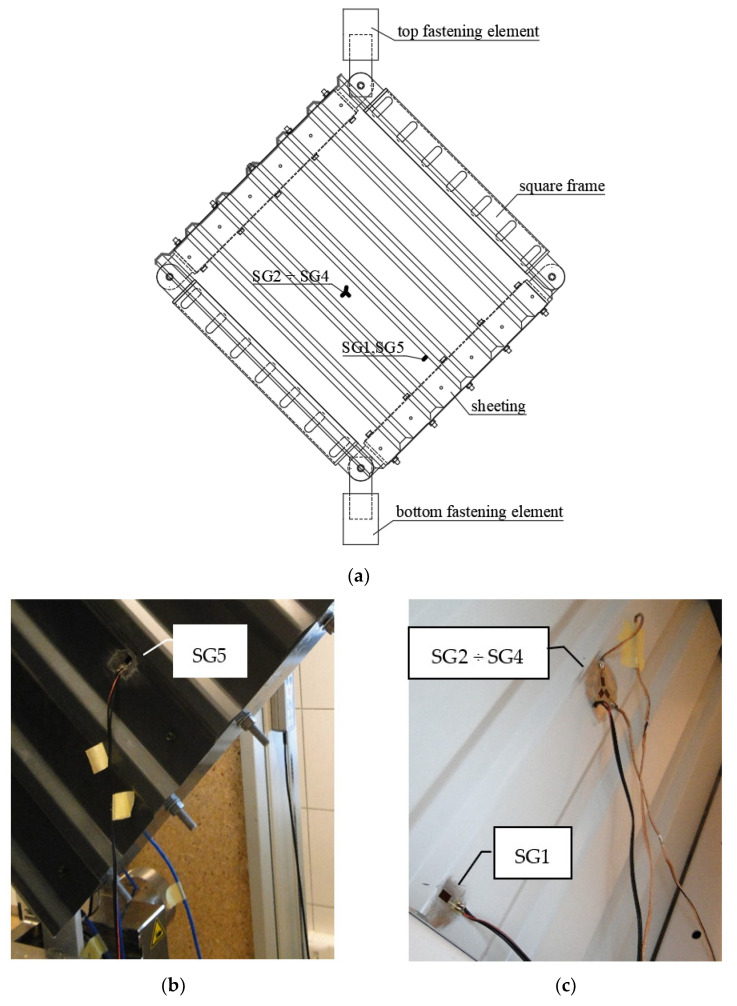
Strain gauge (SG) measurements: (**a**) scheme of the SG location; (**b**) SG 5 during the test; (**c**) SG 1, 2, 3 and 4 during the test.

In nodes 2 and 4 (see Figure 1) extra elements were provided to allow fastening the set-up to the testing machine. These extra elements were totally fixed in both top and bottom fastening element of the testing machine. Next, every extra element was pin-joint in the hinges of the square frame with possible rotation about the axis perpendicular to the plane of the frame; see Figure 3b and Figure 4a. Similar hinges have been constructed in nodes 1 and 3 in Figure 1, as presented in Figure 2. The bottom fastening element was fixed in the initial point during the experiment while the testing machine was inducing displacement of the top fastening element in the vertical direction. Due to pin connection of members, the permanent set-up (square frame) was a mechanism not to resist shear (comparing to the diaphragm stiffness). It allowed us to investigate the resistance and stiffness of shear panels made of corrugated sheeting installed to the frame. Sheet/purlin (or sheet/rafter) fasteners were applied, so flexibility, location and influence of the connection on corrugated sheeting were taken into account. Flexibility of the support elements (purlins, rafters) was included with the use of U-shaped cold-formed plates. The plates were fixed to rigid rectangular hollow section (RHS) profiles in order to fasten the sheeting. Specifications of the applied elements are widely discussed in Section 2.2.

### 2.2. Experimental Model—Specification of the Elements

The model of the experimental set-up is presented in Figure 1; the set-up fastened to the testing machine is presented in Figure 2. The main parts of the permanent experimental set-up were four pin-ended rectangular hollow section profiles (RHS 100 × 4), which formed the mechanism of a square frame (see Figure 1 and Figure 2a). The RHS 100 × 4 profile was assumed rigid enough not to affect the final results. Next the U-shaped cold-formed plates were fastened to the RHS members using bolts as presented in Figure 2b and Figure 3b. The thickness of the U-shaped plate was 0.5 mm; however, in the fastening plane the thickness was increased to 1.5 mm by adding extra plates. The corrugated sheeting is typically fastened to the support structure (e.g., purlin or girder) using self-drilling screws. In order to allow for fastening the corrugated sheeting to the U-shaped plates and for the re-use of the permanent set-up, long holes were designed in the RHS profiles; see Figure 2a. The case of trapezoidal sheeting was investigated, with the height of 18 mm, the length in the direction of the corrugation of 950 mm and the width in the direction parallel to the corrugation of 830 mm. The geometry of the trapezoidal sheeting was chosen according to Eurocode procedures [1,18,19,20], based on the comparison of the value of the ultimate limit state (ULS) combination of loading perpendicular to the plane of the sheeting typical for Poland and the allowable loading for the specimen trapezoidal sheeting. The comparison proved that the space between the supports of the sheeting should not exceed 1 m, while the spacing between support elements in the tests was equal to 0.85 m due to machine limitations. Taking this into consideration, the reduction scale is not such a significant issue. On the other hand, in the experiment only one part of the panel was taken into account; in real structures there are more panels building one roof, which means more complex behaviour. However, in this paper the concentration is primarily on the local effects (support, fasteners) which seem to be crucial in case of parasitic stressed-skin action. In this case, even the reduced scale specimen seems to fulfil its function. The trapezoidal sheeting geometry and the model of the sheet/purlin or sheet/rafter fasteners (self-drilling screws of a diameter 5.5 mm and ethylene propylene diene monomer (EPDM) washer is presented in Figure 3. The screws were used in the centreline of every corrugation.

### 2.3. Experimental Model—Specifications of the Measurements

Experimental analyses were conducted in the testing machine as presented in Figure 1 and Figure 2. The displacement increment over time of the top fastening element of the machine (node 2) was assumed constant. Displacements (node 2), forces (node 4) and time were measured. Additionally, the changes of strains in selected points and directions of the trapezoidal sheeting in relation to the time were registered. The layout of the strain gauges used in the tests is presented in Figure 4. SG 1 and 5 registered the changes of the strains in the direction perpendicular to the corrugations in the side of the middle corrugation, 50 mm from the frame edge (on both sides of the sheeting). SG 2, 3 and 4 formed a rectangular rosette (on one—bottom side of the sheeting) which allowed us to assess principle stresses (assuming elastic modulus *E* = 210 GPa).

Four tests of trapezoidal sheeting panels were conducted. The test 1 assumed the displacement was increasing constantly until the panel failure. In tests 2, 3 and 4 the repeated displacement increase/decrease was implemented. The loading protocol in tests 2, 3 and 4 was as follows:

- Initial force 50 N.
- Increase of the force (displacement increase = constant) until the value of 1000 N—force = constant for 30 s—decrease of the force to the value of 300 N (displacement decrease = constant)—force = constant for 30 s.
- Increase of the force (displacement increase = constant) until the value of 2000 N—force = constant for 30 s—decrease of the force to the value of 300 N (displacement decrease = constant)—force = constant for 30 s.
- Increase of the force (displacement increase = constant) until the value of 3000 N—force = constant for 30 s—decrease of the force to the value of 300 N (displacement decrease = constant)—force = constant for 30 s.
- Increase of the force (displacement increase = constant) until the value of 4300 N—force = constant for 30 s—decrease of the force to the value of 300 N (displacement decrease = constant)—force = constant for 30 s.
- Increase of the force (displacement increase = constant) until the value of 6300 N—force = constant for 30 s—decrease of the force to the value of 300 N (displacement decrease = constant)—force = constant for 30 s.
- Increase of the force (displacement increase = constant) until the failure of the panel.

The initial force in the testing machine was 50 N. Nevertheless, in the initial stage of loading, the loose spaces of the set-up disturbed the measurements (about 3,5 mm and circa 300 N). In the paper diaphragms without this initial stage are analysed.

### 2.4. Numerical Model—General Description

A 3D numerical model of the experimental research was built in ABAQUS software [21]. Geometrically and materially non-linear static analysis was performed. An elastic-plastic model of the material (steel S250GD + Z) was reflected by the following properties: elastic modulus *E* = 210 GPa, Poisson’s ratio *ν* = 0.3 and yield stress *f_y_* = 250 MPa.

**Figure 5:**
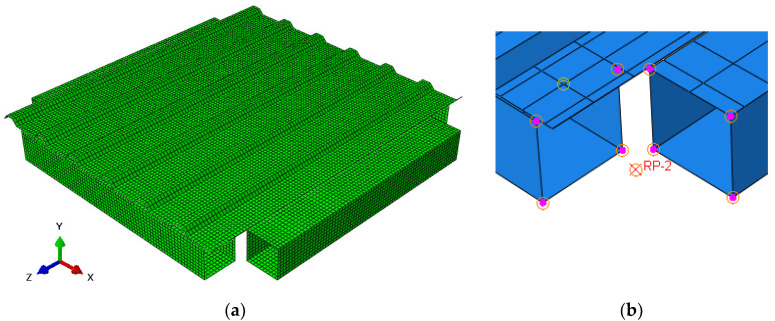
Numerical model: (**a**) view of the whole model with global coordinates; (**b**) detail of the frame hinge.

The frame members and corrugated sheets were modelled by 20,100 shell elements with four nodes and four integration points (S4) and the size of 8–12 mm. Profiles were built with sharp corners (radius of curved elements equal to zero). The meshed structure is presented in Figure 5a.

The frame members were modelled in a simplified way. Each of the RHS profiles, which in tests were the supports for the sheeting, was built as one element together with the U-shaped cold-formed plate. The thickness of the U-shaped plate (0.5 mm on the sides, 1.5 mm on the top) was assigned, so that the stiffness of the profile was mapped. Hinge connections in the frame were included using four reference points (RP in Figure 5b) in four axial nodes of the frame, which were tied (six degrees of freedom fixed) with four corners of the shell frame element. This way allowed us to model the hinge without the necessity of building the details of the connection. The connection is presented in Figure 5b.

Fasteners (self-drilling screws) were mapped using tie connections between nodes (six degrees of freedom fixed). The propping effect was included by modelling the contact between the sheeting and the frame (with the separation allowed after contact).

Boundary conditions were assigned using references points. In nodes 1 and 3 the displacement in y direction was fixed; in node 4—displacements in *x*, *y* and *z* directions were fixed (for numbers of nodes see Figure 1). In node 2 the displacement increase was applied in the direction of the frame diagonal until the failure of the panel. Stress maps were obtained. The numerical analysis results are presented in Section 3.2.

## 3. Results and Discussion

### 3.1. Laboratory Tests

**Figure 6:**
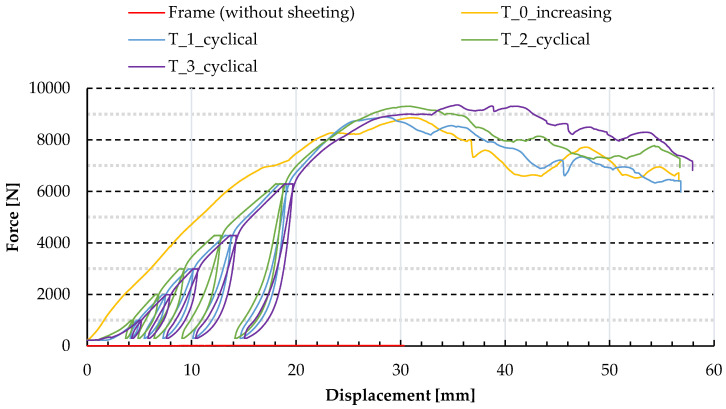
Force–displacement paths obtained in experimental tests.

In the first step of the laboratory analysis, the four-pinned frame of the experimental set-up (see Figure 2a) was tested without the sheeting. The force–displacement outcomes confirmed the assumption that the frame alone, not including trapezoidal sheeting, has a mechanism of negligible stiffness, with regard to shear stiffness of the analysed panel; see Figure 6.

**Figure 7:**
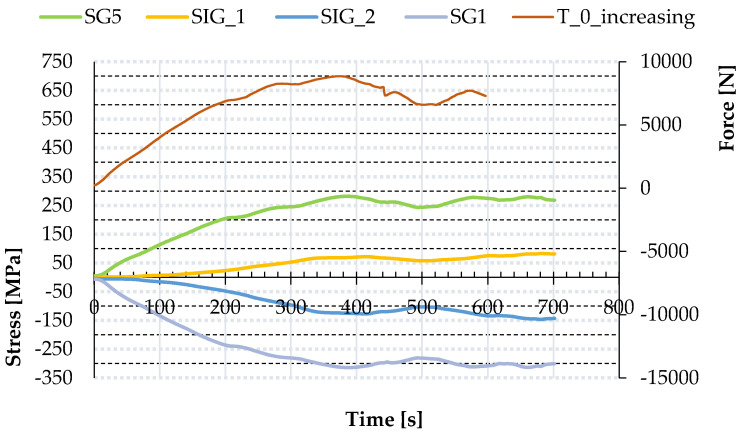
Force–time and stress–time paths obtained in experimental tests for increasing loading.

Next, major tests of trapezoidal sheeting panels were conducted. In the first step, the constant displacement increase was applied leading to panel failure. The force–displacement and force–time path of the main nodes were registered; see “T_0_increasing” in Figure 6 and Figure 7. Moreover, strains were measured by SG (for the location of the SG; see point 2.3), leading to the corresponding stresses: SG1 and SG5—stresses in the direction perpendicular to the corrugation (top and bottom side of the sheeting respectively) and SIG_1 and SIG_2—the principal stresses linked with the outcomes of strain gauge rosette; see Figure 7. Note that the stresses related to profile distortion (in the location of strain gauges SG1 and SG5) start to increase earlier and become much greater than the stresses related to global shear of the panel (in the location of strain gauges SG2, 3, 4). It confirms the prediction that in this variant of panel geometry (relatively high trapezoidal sheeting compared to the panel planar dimensions), the profile distortion dominates the shear strain. What is more, the absolute values of SIG_2 principal stresses were about 1.5–2 times greater than the values of SIG_1 principal stresses. The values were affected by the orthotropy of the trapezoidal sheeting and the location of the SGs on the bottom flange.

**Figure 8:**
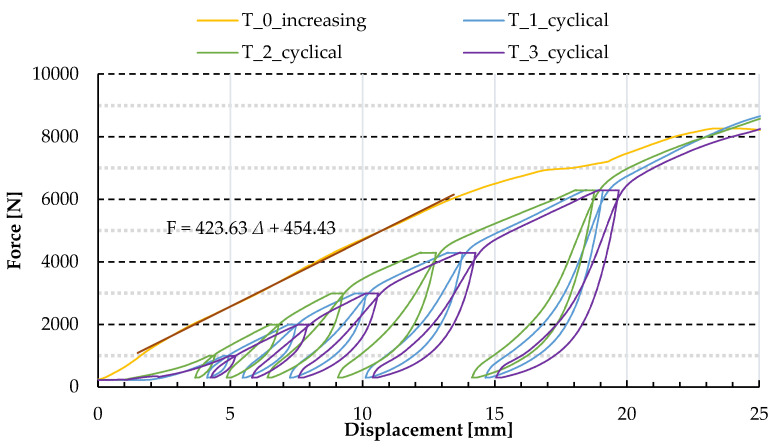
Force–displacement paths obtained in experimental tests—the extract of the outcomes.

In the next step, three cyclic loading tests were conducted on trapezoidal sheeting panels. The diaphragms were loaded six times in every test; each step brought an increasing force level. The last increment led to failure of the panel. The force–displacement path of the main nodes registered by the testing machine is presented in Figure 6 and Figure 8; see “T_1_cyclical,” “T_2_cyclical” and “T_3_cyclical”.

The force–displacement relations corresponding to the repeated loading revealed a wider characteristic of the panel compared to the permanently increasing load (compare T_1-3_cyclical with the T_0_increasing in Figure 8). Hence the diaphragm displays two types of stiffnesses: primary and secondary. When the panel bears a particular force level for the first time, its stiffness is lower (primary stiffness); when the force level is achieved again, after unloading, the stiffness is greater (secondary stiffness). In the course of primary loading, the displacements between elements (e.g., loose space, clearances) are successively removed, some of them permanently. As a result, during reloading the displacement does not reach the level prior to the primary loading. Moreover, stiffness depends on the direction of change which is represented graphically by the hysteresis curves. The area in the centres of hysteresis loops can be interpreted as the energy dissipation mainly due to friction between elements of the panel. The scale of the hysteresis can rely on the rapidity of the loading/reloading.

The trend line for the test with increasing load is shown in Figure 8, where: *F* is force and Δ is displacement. According to the trend line equation, the primary stiffness of the panel is 0.424 kN/mm. The force–displacement relations for increasing and repeated loading make us conclude that both primary and secondary stiffness of the panels in four tests are comparable. Simultaneously, translation of the diagrams is observed. It can arise from different initial clearances in particular panels triggered by assembly imperfections, thus means it can also occur in real structure situation.

**Figure 9:**
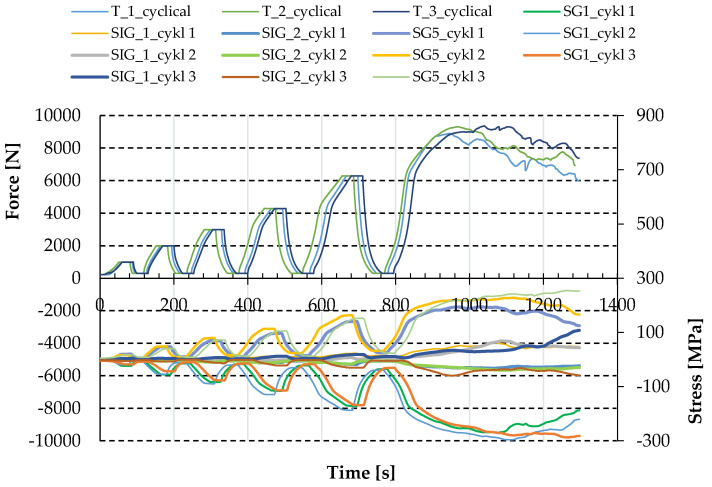
Force–time and stress–time paths obtained in experimental tests for repeated loading.

Similarly to the increasing loading case, in the case of repeated loading strains were also measured by strain gauges (the location of the strain gauges is addressed in point 2.3), leading to stresses: SG1 and SG5—the stresses in the direction perpendicular to the corrugation (top and bottom side of the sheeting respectively); and SIG_1 and SIG_2—the principal stresses in the location of strain gauges rosette, as presented in Figure 9.

After reaching a particular load level, the force was fixed for the time equal to 30 s. During that time, the displacements were increasing while the force and the stresses in the sheeting remained constant; see Figure 8 and Figure 9. It can signalise the local plasticity of the trapezoidal sheeting, e.g., in the area of screws and the longitudinal fold lines of the sheeting—even in the case of load increments when the stresses (indirectly) measured by strain gauges SG1 and SG5 in the area close to the support were below the yield stress.

Permanent displacements increased at every loading; the stresses followed; see Figure 9. That confirms the theory of local plasticity of the trapezoidal sheeting, which can lead to leakage or even failure of the sheeting.

**Figure 10:**
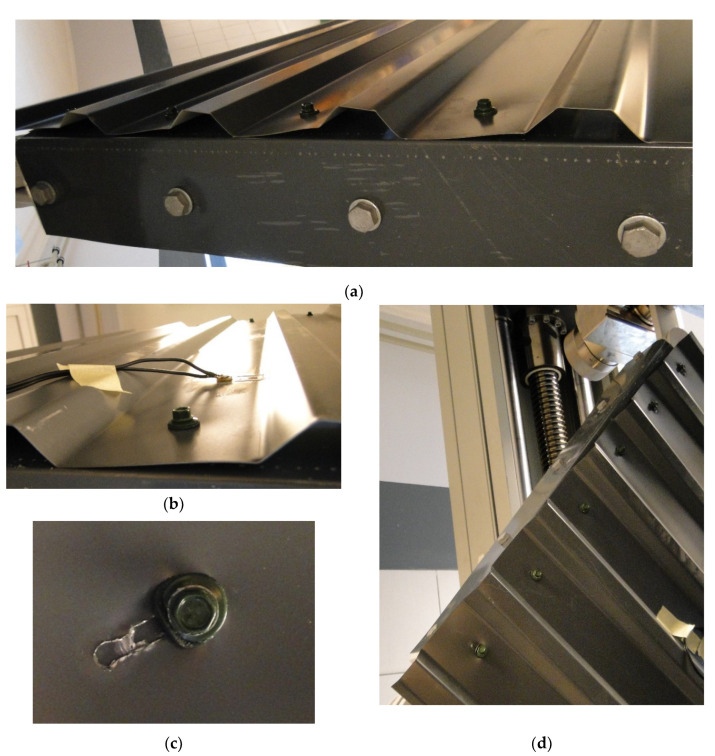
Deflections of trapezoidal sheeting observed during laboratory tests: (**a**) profile distortion—global view; (**b**) profile distortion—local view; (**c**) hole elongation; (**d**) rotation of the sheeting due to hole elongation.

Figure 10 shows deflection modes observed during tests. First of all, profile distortion occurs; next comes hole elongation, leading to the rotation of the sheeting. Hole elongation due to plasticity of the sheeting turned out to be a crucial failure mechanism in this series of tests.

At the first stage of the unloading, both displacements and stresses decrease rapidly (small decrease of the displacements means that the structure is rigid at the beginning of the uploading). Visual observations confirm that the profile distortion decreases significantly. It is interpreted as the fact that in first unloading stage the traction between elements supresses the displacements between members and the stiffness is mostly influenced by taking back the profile distortion.

### 3.2. Numerical Analysis

**Figure 11:**
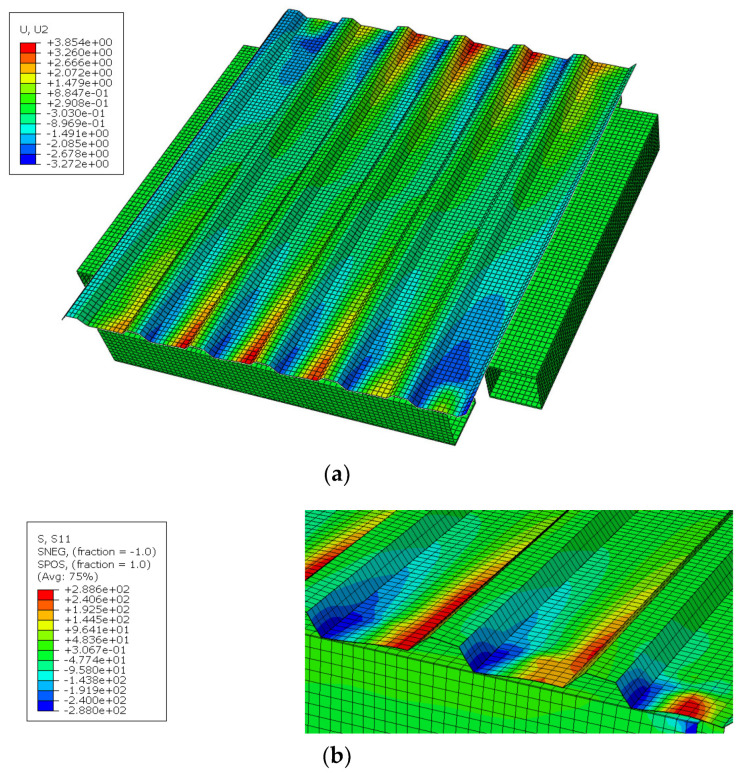
Selected results of the numerical analysis: (**a**) displacements in the direction perpendicular to the panel [mm]; (**b**) stresses in the direction perpendicular to the corrugation [MPa].

**Figure 12:**
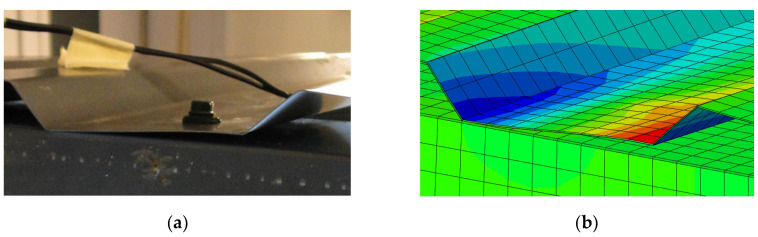
The comparison of the shape of the profile deformation: (**a**) test, (**b**) numerical model.

**Figure 13:**
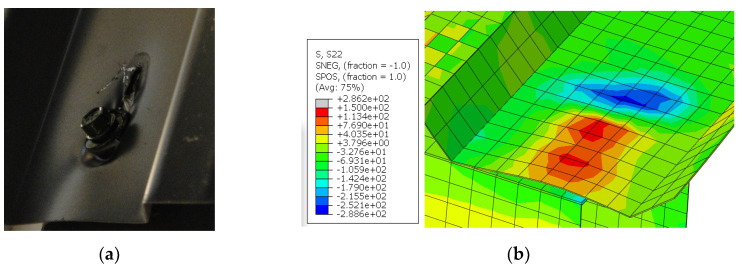
The comparison of the results in the area of self-drilling screw: (**a**) test, (**b**) numerical model (stresses in the direction parallel to the corrugation [MPa]).

The 3D numerical model of the experiment was run in ABAQUS software [21]. In numerical estimations, the structure was built in simplified way, as was described in detail in Section 2.4 (screws as tie connections between two nodes, no holes, profiles with sharp corners, only increasing force considered). As a result, the numerical stiffness was greater than experimental stiffness and the effect of primary/secondary stiffness (which arises from, among others, hole elongation, natural spacing between assembled element—parameters not mapped in the numerical simulations) was not captured. However, other selected results—displacements in the direction perpendicular to the panel and stress map in the direction perpendicular to the corrugation (the direction of the strain gauges SG1 and SG5)—are presented in Figure 11. The results confirmed the dominant character of profile distortion (shape of the profile deformation, stress distribution in the area of the support, opposite signs of the stresses on both sides of the sheeting). The shapes of profile deformation are compared in Figure 12. The results in the area of screw fasteners are compared in Figure 13.

## 4. Conclusions

Experimental tests on a small-scale shear panel made of trapezoidal sheeting were conducted in order to observe the behaviour of the diaphragm under increasing and repeated load. The force–displacement relation (in a testing machine) and the strains in selected areas of the sheeting (using strain gauges) were measured. The results revealed the hysteresical character of panel work and occurrence of persistent deflections and stresses, which remain even after the unloading and suggest invisible plastic strains in the panel. In the case of fatigue accumulation, the latter may lead to the leakage, even failure.

The experimental results showed the character of work of the cladding in case of stressed-skin effect under repeated load (primary/secondary stiffness), the important phenomena and the failure mechanism, especially in the support area of the panels. The outcomes show the relation between the stresses, force–displacement paths and the mechanism of failure, which potentially can be used in the monitoring of existing buildings in order to assess parasitic stressed-skin action in a non-destructive way. What is more, roof rigidity determined by measurements may be incorporated in metal sheet design to determine the flexibility and stiffness of connections.

The results give a basis to the non-destructive testing of the existing structures. By knowing the displacement ranges of the existing structure panels (e.g., using inductive sensors), it seems that the condition of the structure can be assessed (also for structures which were designed without consideration of the stressed-skin effect) by the comparison of the results with the data obtained in corresponding laboratory tests. What is more, measurements which record the loading–unloading cycles and even cycles of loading in opposite directions gives the possibility of obtaining displacement paths in order to access the hysteresis character of work of the existing structure. If the hysteresis is significant even in the range of the typical loading conditions, it suggests that the structure adjusts to the loading in non-linear way, which means potentially dangerous situation—correction or redesigning of the structure should be considered. Registering of the displacement of the panels in a real structure can be complemented by registering the strains using the strain gauges in the areas predicted as crucial, e.g., near the support of the panel, which would help to access the condition of the sheeting and even the support elements.

There are still some factors which were not included in this study and are planned for the next test series or require further analysis:

- Further experimental series, especially with other schemes of the screws, with two sheets of covering with/without seam fasteners, with thermal insulation between sheeting and the support element, etc.
- Improvement of the numerical model in terms of repeated loading and stiffness estimations.
- The analysis of the influence of the test speed on the hysteresis loop.
- The reduction scale in cases of higher profiles of the sheeting.
- The modification of the experimental set-up so that another failure mechanism could be achieved.

## References

[1] EN 1993-1-3:2006 (2006). Eurocode 3. Design of Steel Structures. Part 1-3: General Rules. Supplementary Rules for Cold-Formed Members and Sheeting.

[2] (1995). European Convention for Constructional Steelwork—TC7, TWG 7.5. European Recommendations for the Application of Metal Sheeting Acting as a Diaphragm. Stressed Skin Design.

[3] Lendvai A., Joó A., Dunai L. (2018). Experimental full-scale tests for development of diaphragm action—Part I.—Experimental results. Thin-Walled Struct..

[4] Lendvai A., Joó A., Dunai L. (2018). Experimental full-scale tests for development of diaphragm action—Part II.—Effect of structural components on shear flexibility. Thin-Walled Struct..

[5] Lendvai A., Joó A. (2019). Improvement of stressed skin design procedure based on experimental and numerical simulations. J. Constr. Steel Res..

[6] Wrzesien A.M., Lim J.B.P., Xu Y., MacLeod I.A., Lawson R.M. (2015). Effect of stressed skin action on the behaviour of cold-formed steel portal frames. Eng. Struct..

[7] Korcz N., Urbańska-Galewska E. (2018). Influence of sheet/purlin fasteners spacing on shear flexibility of the diaphragm. MATEC Web Conf..

[8] Korcz N., Urbańska-Galewska E. (2018). Influence of fasteners and connections flexibility on deflections of steel building including the stressed skin effect. Tech. Sci..

[9] Davies J.M. (2006). Developments in stressed skin design. Thin-Walled Struct..

[10] Nagy Z., Pop A., Mois I., Ballok R. (2016). Stressed skin effect on the elastic buckling of pitched roof portal frames. Structures.

[11] Gryniewicz M. (2018). Metoda Modelowania Konstrukcji Hal Stalowych Obudowanych Blachą Trapezową. Ph.D. Thesis.

[12] Bakhti F., Tremblay R., Rogers C.A. Revisiting the SDI and ECCS methods for in-plane shear flexibility of metal roof deck diaphragms using 3D non-linear finite element analysis. Proceedings of the 15th World Conference on Earthquake Engineering—15WCEE.

[13] Rzeszut K., Gastecki A., Czajkowski A. Parameter identification in FEM models of thin-walled purlins restrained by sheeting, Recent advances in computational mechanics. Proceedings of the 20th International Conference on Computer Methods in Mechanics (CMM 2013).

[14] Davies J.M., Roberts M.J., Wang Y.C. Recent developments in stressed skin theory. Proceedings of the 8th International Conference on Thin-Walled Structures—ICTWS.

[15] Davies J.M., Roberts M.J., Wang Y.C. The testing and analysis of stressed skin diaphragms. Proceedings of the 8th International Conference on Thin-Walled Structures—ICTWS.

[16] Davies J.M., Roberts M.J., Wang Y.C. Stressed-skin action in sandwich panel roofs. Proceedings of the 9th International conference on Steel and Aluminium Structures—ICSAS19.

[17] Furtado A., Rodrigues H., Arêde A., Varum H., Grubišić M. (2018). Prediction of the earthquake response of a three-storey infilled RC structure. Eng. Struct..

[18] Polish Committee for Standarization (2004). PN-EN 1990:2004 Eurocode 0. Basis of Structural Design.

[19] Polish Committee for Standarization (2008). PN-EN 1991-1-3:2005 Eurocode 1. Actions on Structures. Part 1-3: General Actions. Snow Loads.

[20] Polish Committee for Standarization (2005). PN-EN 1991-1-4:2008 Eurocode 1. Actions on Structures. Part 1-4: General Actions. Wind Actions.

[21] ABAQUS (2008). Theory Manual.

